# Distinct transcriptional responses of lymphatic endothelial cells to VEGFR-3 and VEGFR-2 stimulation

**DOI:** 10.1038/sdata.2017.106

**Published:** 2017-08-29

**Authors:** Lothar C. Dieterich, Luca Ducoli, Jay W. Shin, Michael Detmar

**Affiliations:** 1 Institute of Pharmaceutical Sciences, Swiss Federal Institute of Technology (ETH) Zurich, 8093 Zurich, Switzerland; 2 RIKEN Center for Life Science Technologies, Division of Genomic Technologies, Yokohama, Kanagawa 230-0045, Japan

**Keywords:** Growth factor signalling, Transcriptomics

## Abstract

Vascular endothelial growth factors (VEGFs) and their receptors play crucial roles in the formation of blood and lymphatic vessels during embryogenesis, and also under pathologic conditions in the adult. Despite intensive efforts over the last decades to elucidate the precise functions of VEGFs, transcriptional responses to VEGF receptor stimulation are still not fully characterized. To investigate the specific transcriptional effects of VEGFR-2 and VEGFR-3 activation, we performed a correlation analysis of previously published CAGE sequencing and microarray data of human lymphatic endothelial cells (LECs) stimulated with distinct VEGFs acting through either VEGFR-2 or VEGFR-3. We identified that specific activation of VEGFR-3 by VEGF-C156S results in the downregulation of many genes involved in immune regulation and inflammation, suggesting that VEGFR-3 stimulation has direct anti-inflammatory effects. Comparing CAGE and microarray data sets, we furthermore identified a small number of genes that showed a receptor-dependent response in LECs, demonstrating that these receptors, despite activating very similar signaling pathways, fulfill overlapping but not identical functions within the same cell type (LECs).

## Introduction

The vascular system, consisting of blood and lymphatic vessels, is crucially dependent on vascular endothelial growth factors (VEGFs) and their receptors (VEGFRs), which regulate the proliferation and function of endothelial cells. In humans, the VEGF family comprises five ligands, placenta growth factor (PLGF), VEGF-A, VEGF-B, VEGF-C and VEGF-D, that are structurally related and predominantly present as homodimers in their mature form^1^. VEGFs exert their function by binding to the three known VEGFRs, VEGFR-1, VEGFR-2 or VEGFR-3, which are expressed by blood vessel endothelial cells (BECs) in case of VEGFR-1 and -2, and lymphatic vessel endothelial cells (LECs) in case of VEGFR-2 and -3. Some expression of VEGFR-3 in angiogenic blood vessels has been described as well^2^. In addition, VEGFs bind to co-receptors such as neuropilins (NRP) 1 and 2 as well as heparan sulfate proteoglycans, that do not signal directly but modulate the interaction between VEGFs and VEGFRs and thereby affect VEGF signaling indirectly^1^.

The individual members of the VEGF family display selective binding affinities for one or several VEGFRs. PLGF and VEGF-B exclusively interact with VEGFR-1. VEGF-A, the most potent inducer of endothelial responses within the family, binds to both VEGFR-1 and VEGFR-2. Although the affinity of VEGF-A is higher for VEGFR-1 than for VEGFR-2, the latter receptor has a much higher kinase activity and is thus regarded as the most important receptor for transmitting the effects of VEGF-A. VEGF-C and VEGF-D differ from the other ligands of the family in that they are produced as immature precursor proteins, requiring proteolytic maturation in order to gain their full activity, and preferentially bind to VEGFR-3. In their fully mature form, however, both ligands also act as VEGFR-2 agonists^3^. Of note, a mutated form of VEGF-C, in which Cys^156^ is replaced by a Ser residue (VEGF-C156S), displays reduced affinity for VEGFR-2, and has thus been regarded as a selective VEGFR-3 stimulator^4^.

Signaling through VEGFR-2 is initiated upon ligand binding that stabilizes the receptor in a dimeric state and induces conformational changes, activating the intracellular kinase domains^5^. This in turn leads to the phosphorylation of specific Tyr residues in the cytoplasmic tail of the receptor, which serve as docking sites for adapter proteins needed to initiate multiple downstream signaling cascades such as the PI3K-AKT, PLC-γ and Ras-Raf-ERK signaling pathways. The biochemical processes immediately downstream of receptor activation have been investigated to considerable detail (reviewed in ref. 1). On the one hand, these signaling cascades trigger immediate, transcription independent cellular responses such as cell migration and opening of cellular junctions. On the other hand, VEGFR signaling activates several transcription factors (TFs), including AP-1, NFAT and FOXO family members, that regulate several genes involved in cell proliferation, differentiation and survival. Compared to VEGFR-2, the VEGFR-3 signaling cascade is somewhat less well understood but is regarded to activate very similar pathways as those downstream of VEGFR-2 (ref. 1). Nonetheless, the changes in gene expression downstream of the two receptors have not been fully characterized.

Using CAGE RNA sequencing^6^ in conjunction with the FANTOM5 project^7^, we recently analyzed transcriptional changes elicited by VEGF-C156S mediated VEGFR-3 stimulation in primary human lymphatic endothelial cells (LECs) and identified several TFs involved in this response. The majority of these TFs are well known ‘immediate early’ TFs that are induced upon signaling through various receptor tyrosine kinases such as EGR, FOS and JUN. Importantly, we also identified TFs with LEC-specific functions during VEGFR-3 signaling. For example, the homeobox TF HOXD10 that is constitutively present in LECs but not in BECs, is required for the induction of a second, downstream TF, namely NR4A1 (ref.  8). Another TF, MAFB, was induced and activated in LECs after VEGFR-3 and VEGFR-2 stimulation but not in BECs after VEGFR-2 stimulation, indicating that in the context of VEGF signaling, MAFB is a LEC-specific TF^9^. These data indicate that there are differences in TF activation downstream of VEGFRs, depending on the endothelial cell type, that are likely to result in the induction of different target genes in LECs and BECs. It is unclear, however, whether there also are receptor-specific differences in the regulation of target genes downstream of VEGFR-2 stimulation compared to VEGFR-3 stimulation, and whether these differences are cell type-dependent.

Our previous analysis of the CAGE RNA sequencing data only revealed upregulated but not downregulated genes after VEGF-C156S treatment of LECs^9^. Here, we re-analyzed the same data set using a different analysis method (DESeq2). We identified multiple downregulated genes that were frequently associated with pro-inflammatory functions. Furthermore, we performed a combined analysis of the CAGE sequencing data and our previously published microarray-based gene expression data of primary human LECs treated with VEGF-A or fully mature VEGF-C, resulting in activation of either VEGFR-2 or of both VEGFR-2 and VEGFR-3, respectively^10^. We found that even though the majority of target genes are comparably regulated by VEGFR-2 and VEGFR-3 activation in human LECs, a small number of distinct genes showed receptor-specific responses within the same cell type.

## Results

### VEGF-C156S provokes complex changes of LEC gene expression

**Figure 1:**
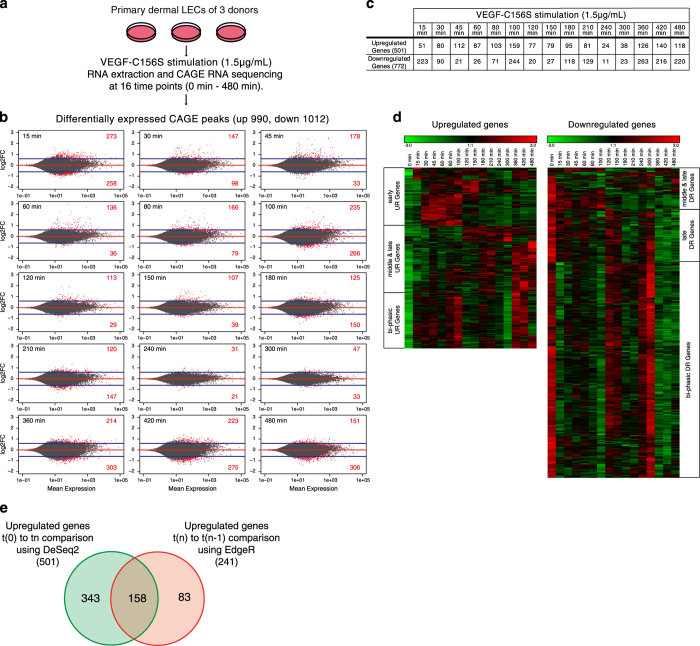
Characterization of transcriptional changes in primary human LECs treated with VEGF-C156S by CAGE RNA sequencing. (**a**) Schematic overview of the experimental procedure: Primary human dermal LECs from three individual donors were incubated with VEGF-C156S (1.5 μg/ml). RNA was extracted at 16 different time points from 0 min (baseline) to 480 min and subjected to CAGE RNA sequencing. (**b**) MA plots showing the log_2_FC compared to the expression level derived from DESeq2 analysis of differentially expressed CAGE peaks at each time point compared to the baseline. Red dots represent significantly altered CAGE peaks (*P*<0.05) and horizontal blue lines represent the log_2_FC cutoff of 0.6 resp. −0.6. The red numbers in the plots correspond to the number of significantly altered CAGE peaks above the log_2_FC cutoff. (**c**) Summary table of the number of genes corresponding to the DE CAGE peaks. (**d**) Heat map based on the expression levels (tpm) of all genes corresponding to DE CAGE peaks after hierarchical clustering. Colors code for log_2_FC values on a scale from −3 to +3. (**e**) Venn diagram comparing the upregulated genes between DESeq2 and previously published EdgeR analyses.

We previously published a CAGE RNA sequencing data set of primary human dermal LECs treated with the VEGFR-3-specific ligand VEGF-C156S, analyzed at 16 different time points from 0 min (control) to 480 min (ref. 9) (Fig. 1a). In this study, we had analyzed differential gene expression by comparing each time point t_n_ to the preceding time point t_n-1_ using the EdgeR method^11^. Since this analysis likely failed to identify genes with small, gradual changes in gene expression, we re-analyzed the data set comparing each time point t_n_ to the baseline time point t_0_ using paired DESeq2 (ref. 12). Among 85774 unique CAGE peaks expressed in the data set, we identified a total of 990 upregulated (*P*<0.05, log_2_ fold change (log_2_FC)>0.6) and 1012 downregulated (*P*<0.05, log_2_FC<−0.6) peaks (Fig. 1b). Activation of VEGFR-3 with VEGF-C156S caused two ‘waves’ of CAGE peak upregulation, the first from 15 to 210 min, and a second one from 360 to 480 min. Downregulated CAGE peaks were most frequently detected at the 15 and 100 min time points and during the late phase of the time course (360 to 480 min), corresponding to the second wave of upregulated CAGE peaks (Fig. 1b).

On the gene level, these differentially expressed (DE) CAGE peaks corresponded to 501 upregulated and 772 downregulated genes, according to the current CAGE peak annotation (Fig. 1c and ‘Upregulated Genes after VEGFC156S stimulation’, ‘Downregulated Genes after VEGFC156S stimulation’, Data Citation 1). Hierarchical clustering analysis of the upregulated genes over the 16 time points showed three distinct gene clusters: genes upregulated at the early time points (15 to 100 min), genes upregulated at the middle to late time points (120 to 480 min), and genes with a ‘bi-phasic’ upregulation from 60 to 100 min and from 360 to 480 min. Similarly, the downregulated genes also clustered into three groups: genes downregulated in both the middle and late phase of the time course (120 to 480 min), genes downregulated only in the late phase (360 to 480 min) and genes with a bi-phasic downregulation (15 to 100 min and 360 to-480 min) (Fig. 1d). Furthermore, we compared the 501 upregulated genes identified here with the 241 upregulated genes found in our previous analysis^9^. Among the 158 overlapping genes, we found several important lymphatic TFs such us KLF4, SOX18, and MAFB (Fig. 1e). Interestingly, using DESeq2, we found upregulation of further TFs such as FOXC2, ELK1, and NFATC1 which have previously been associated with vascular development and / or angiogenesis^13–15^. Taken together, these results indicate that selective activation of VEGFR-3 causes complex changes in gene expression in LECs, with multiple target genes showing distinct kinetics of up- or downregulation.

### Gene ontology analysis reveals downregulation of immune response-related genes after VEGFR-3 stimulation

**Figure 2:**
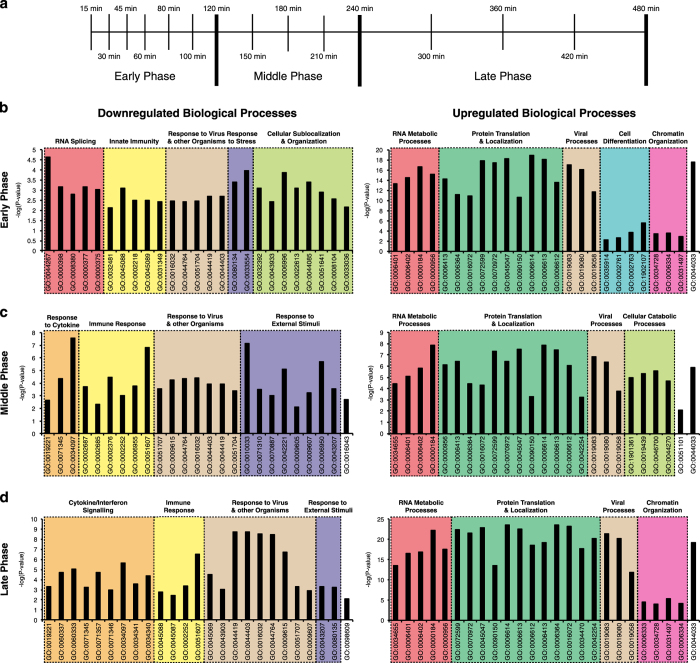
Gene ontology analysis and clustering of up- and downregulated genes. (**a**) DE genes were grouped into three distinct phases of the time course experiment: early (15 to 120 min), middle (150 to 240 min), and late phase (300 to 360 min). (**b**–**d**) Gene ontology (GO) analysis and clustering of the 25 most highly enriched GO biological process terms into related topics for the early (**b**), middle (**c**) and late (**d**) phase of the time course. GO analysis of downregulated genes is shown in the left side panels, GO analysis of upregulated genes is shown in the right side panels.

To characterize the transcriptional changes elicited by VEGFR-3 activation in LECs functionally, we grouped the differentially expressed genes into 3 groups, roughly corresponding to the clusters observed before: early phase genes (15 to 120 min), middle phase genes (150 to 240 min) and late phase genes (300 to 480 min) (Fig. 2a). Then, we performed a gene ontology (GO) analysis of the up- and downregulated genes in each of these phases, considering the ‘biologic process’ GO terms (Fig. 2b–d and ‘GO_BP analysis’, Data Citation 1). Strikingly, we found that downregulated genes were enriched for GO terms related to immune regulation and cytokine (particularly interferon) responses, indicating that VEGFR-3 stimulation has a direct, anti-inflammatory effect on LECs. Downregulated genes included adhesion molecules (SELE, VCAM1), cytokines and chemokines (CXCL10, CXCL11) and several INF-γ related genes (IRF2, IRF3, IFIH1, IFI16, IFI35, IFIT3). In line with our previous analysis^9^, the upregulated genes were enriched for GO terms related to chromatin organization, mRNA expression, and protein translation and localization (Fig. 2b–d). We found an enrichment of RNA splicing-associated genes among the early downregulated genes, whereas the upregulated genes were enriched for cell differentiation-associated genes (Fig. 2b). Similar results were obtained when ‘molecular function’ GO terms were used (‘GO_MF analysis’, Data Citation 1). Taken together, these results indicate that VEGFR-3 stimulation results in the upregulation of genes associated with gene expression and protein translation, whereas genes involved in immune regulation and cytokine (interferon) responses are downregulated.

### Comparison of VEGF-A and VEGF-C156S induced gene expression reveals a small set of receptor-specific target genes

**Figure 3:**
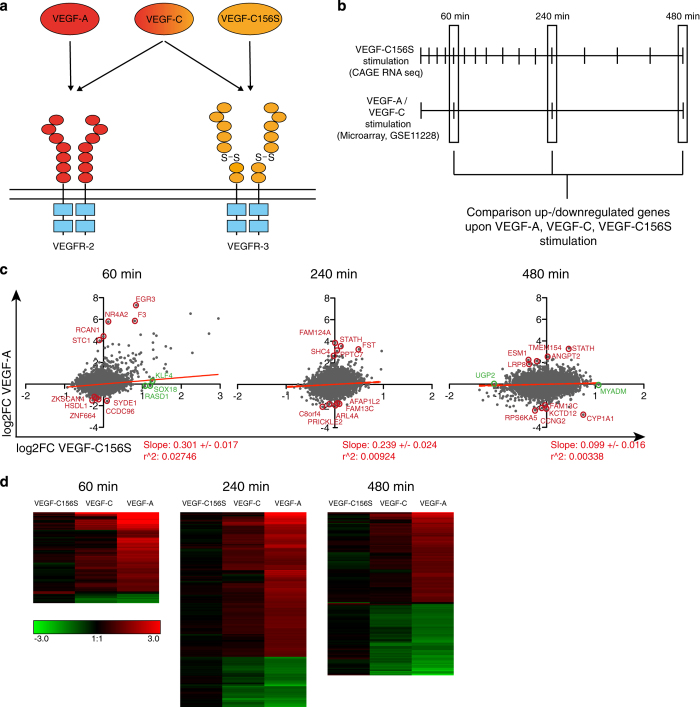
Differential gene regulation by VEGFR-2 and VEGFR-3 in LECs. (**a**) Schematic representation of the binding abilities of VEGF ligands to VEGFR-2 and -3. VEGF-A binds to VEGFR-2 exclusively, whereas fully mature, wildtype VEGF-C can act on both VEGFR-2 and VEGFR-3. VEGF-C with a Cys^156^=>Ser mutation acts as a selective VEGFR-3 agonist. (**b**) Schematic illustration of the three time course gene expression data sets used for the analysis. CAGE RNA sequencing of LECs stimulated with VEGF-C156S was performed at 16 different time points over a course of 480 min. The 60, 240 and 480 min time points were compared with previously published microarray based gene expression data of LECs stimulated with VEGF-A or mature wildtype VEGF-C. (**c**) Correlation of log_2_FC of all matchable genes induced by VEGF-A (y-axes) and VEGF-C156S (x-axes) in LECs at the 60, 240 and 480 min time point. Linear regression and Pearson correlation coefficients are indicated in red. The most differentially regulated genes (up to 5) are indicated in dark red (genes up- or downregulated by VEGF-A but not by VEGF-C156S) and green (genes up- or downregulated by VEGF-C156S but not by VEGF-A). (**d**) Genes were considered as differentially regulated by VEGF-C156S and VEGF-A if they were strongly regulated by at least one of the ligands (*P*<0.05, log_2_FC>1 or <−1), and if the difference in log_2_FC values after VEGF-A and VEGF-C156S stimulation was at least four-fold. The heat maps show the log_2_FC values of all differentially regulated genes after VEGF-C156S, wildtype VEGF-C and VEGF-A stimulation.

VEGF-C156S is a selective agonist of VEGFR-3 (ref. 4), whereas VEGF-A mainly acts through VEGFR-2, and mature wildtype VEGF-C triggers both receptors (Fig. 3a). We previously published microarray-based (ABI Human Genome Survey V2.0) gene expression data of primary dermal LECs stimulated with VEGF-A or mature wildtype VEGF-C (Data Citation 2)^10^, 60 min, 240 min, 480 min and 24 h after stimulation. We decided to use the VEGF-A data to identify genes regulated by either VEGFR-2 or VEGFR-3 stimulation, comparing them to the VEGF-C156S stimulation time course at the three overlapping time points 60 min, 240 min, and 480 min (Fig. 3b). To this end, we correlated the log_2_FC values of each gene after VEGF-A or VEGF-C156S stimulation with each other (Fig. 3c). The correlation was relatively low, especially at the two later time points, probably due to technical biases between CAGE RNA sequencing and the microarrays. Correspondingly, we observed a similarly low correlation when we compared VEGF-C156S with wildtype VEGF-C induced gene expression, but a much higher correlation when we compared VEGF-A with wildtype VEGF-C induced gene expression (Supplementary Fig. 1). Nonetheless, at the 60 min time point, a positive correlation between the VEGF-C156S and VEGF-A data sets was observed, indicating that the majority of target genes are similarly regulated by VEGFR-2 and VEGFR-3 stimulation. However, we also noted several ‘outlier’ genes, which appeared to be regulated much more (or even exclusively) by either VEGF-C156S or by VEGF-A, and could thus represent receptor-specific target genes.

To analyze differences between the VEGF-A induced and VEGF-C156S induced target genes in an unbiased way, we selected candidate genes that were significantly and strongly regulated by at least one of the ligands (*P*<0.05, log_2_FC>1 or <−1), and that showed at least a 4-fold difference in their log_2_FC induced by VEGF-A and VEGF-C156S. We identified 108 differentially regulated (DR) genes at the 60 min time point, 232 at the 240 min time point, and 194 at the 480 min time point (Fig. 3d and ‘Genes differentially regulated by VEGF-A and VEGF-C156S’, Data Citation 1). The vast majority of these were specifically regulated by VEGF-A / VEGFR-2 (the 5 most specific genes are highlighted in red in Fig. 3c) and included well-known VEGF-A target genes such as ESM1, ANGPT2 and RCAN1 (refs 16–19). We also identified a small group of genes specifically regulated by VEGF-C156S (highlighted in green in Fig. 3c). Interestingly, these genes included two important TFs in LEC differentiation and biology, SOX18 and KLF4 (refs 9,20,21).

### RCAN1, ANGPT2, and ESM1 are selectively induced by VEGF-A/VEGFR-2 signaling

**Figure 4:**
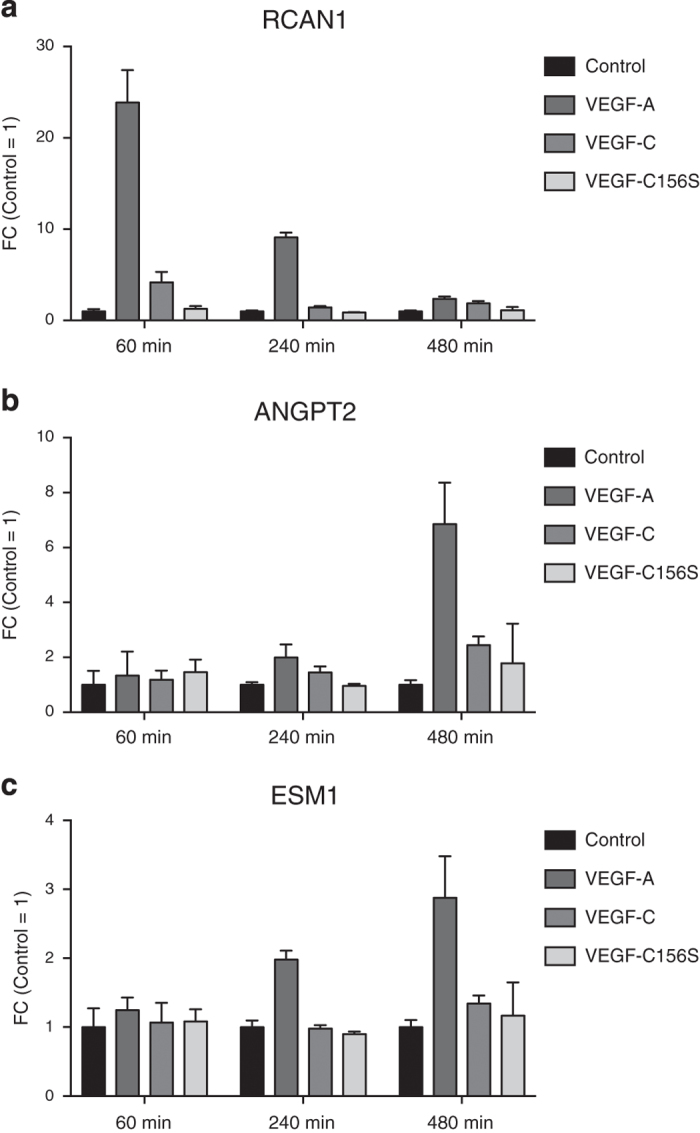
RCAN1, ANGPT2 and ESM1 are selectively regulated by VEGF-A/VEGFR-2 signaling. (**a**–**c**) Validation of the selective upregulation of (**a**) *RCAN1*, (**b**) *ANGPT2* and (**c**) *ESM1* by VEGF-A (20 ngml^−1^) but not by mature wildtype VEGF-C (500 ngml^−1^) or VEGF-C156S (1.5 μgml^−1^) using qPCR (one representative experiment is shown).

To confirm the selective upregulation of RCAN1, ANGPT2, and ESM1 by VEGF-A/VEGF-R2 signaling, we quantified the gene expression of these genes after VEGF-A (20 ngml^−1^), wildtype VEGF-C (500 ngml^−1^) and VEGF-C156S (1.5 μgml^−1^) incubation at 60, 240 and 480 min, using qPCR. Consistent with our *in silico* results, RCAN1 was selectively induced at the 60 min time point (Fig. 4a), and ESM1 and ANGPT2 at the 480 min time point, after VEGF-A stimulation (Fig. 4b,c).

## Discussion

Activation of VEGFR-3 results in various cellular responses in lymphatic endothelial cells, including cell migration, proliferation and changes in the intercellular junctions, which affects monolayer permeability^22–24^. Some of these cellular effects occur within minutes after receptor stimulation and are therefore considered to be independent of *de novo* gene expression, whereas others clearly require a transcriptional response. We recently characterized this transcriptional response of primary human LECs during the first 8 h after treatment with the VEGFR-3 specific ligand VEGF-C156S using CAGE RNA sequencing, which allowed us to identify several TFs involved in the induction of downstream genes^8,9^. However, we initially did not identify any downregulated genes. Changing the *in silico* analysis approach as described here, we now discovered a large number of downregulated genes based on the same CAGE RNA sequencing data set. Likely, the observed differences in the analysis results are due to 3 main reasons: First, we used a different analysis algorithm, namely DESeq2 (ref. 12), as compared to EdgeR^11^ used previously. Both DESeq2 and EdgeR are commonly employed for the analysis of RNA sequencing data but differ in various crucial aspects, including the normalization method and the way how the data dispersion is estimated^25^. Consequently, side-by-side comparison of EdgeR and DESeq2 resulted in overlapping, but not identical sets of DE genes in previous studies^25–27^. As of now, there is no consensus which of the two methods should be used preferentially. However, one study found that DESeq2 is slightly more conservative than EdgeR, meaning that the rate of true positive results, but also the rate of type I errors (false positives) are lower^26^. This suggests that the increased number of DE genes identified here is not simply due to false positives. Secondly, we used a different scheme to calculate the contrasts, comparing each time point t_n_ of the stimulation with the baseline t_0_ (0 min), whereas previously, t_n_ was compared to t_n-1_, which probably resulted in type II errors (false negatives) in case of genes with small and/or gradual changes in expression. Finally, using a multi-factorial design, we now adjusted for differences between the 3 individual LEC donors, which conceivably increased the statistical power of our analysis.

In agreement with our previous analysis^9^, the new analysis confirmed that the genes upregulated during the first hours after VEGFR-3 stimulation are to a large extent associated with transcription-related processes, including chromatin organization, RNA synthesis and stability. In addition to our previous analysis, we identified further TFs induced by VEGF-C156S, including FOXC2, ELK1, and NFATC1, which have been reported to regulate vascular development and angiogenesis before^13–15^. Importantly, our data also indicate that VEGFR-3 activation exerts direct anti-inflammatory effects on LEC, by downregulation of several immune- and cytokine-response associated genes. This is in line with the reported anti-inflammatory effects of VEGF-C on macrophages^28,29^ and indicates that VEGF-C, that has previously been found to reduce inflammation when applied *in vivo*^30–32^, not only acts via an increase of the lymphatic drainage, but also by inhibiting inflammatory signaling in LEC. Interestingly, these anti-inflammatory effects of VEGF-C are reminiscent of the effects of VEGF-A signaling in BEC^33,34^.

VEGFR-2 and VEGFR-3 are structurally related and considered to activate similar intracellular signaling cascades^1^. Nonetheless, VEGF-A and VEGF-C have been described to elicit different effects on lymphatic vessels *in vivo*, although the literature is not entirely consistent in this regard. For example, adenoviral delivery of VEGF-A into the ear of mice has been reported to induce massive dilation of lymphatic vessels^35^, whereas others found lymphatic dilation and formation of new lymphatic vessels only after delivery of VEGF-C, but not of VEGF-A^36–38^. This prompted us to attempt to identify genes differentially regulated by VEGFR-2 and VEGFR-3 within the same cell type, namely primary human LECs, by comparing our CAGE RNA sequencing data set of VEGF-C156S stimulated LECs with a microarray study of VEGF-A and wildtype VEGF-C stimulated LECs previously published by our group^10^. Naturally, cross-platform comparison of gene expression is problematic, due to inherent technical biases. Many studies have described a relatively good correlation between conventional RNA sequencing and microarray results obtained from the same starting material^39–41^. Similarly, CAGE RNA sequencing, in which only short (ca. 20 nt) 5’ fragments of mature, capped RNAs are analyzed, has been found to correlate well with conventional RNA sequencing^42^. Nonetheless, CAGE RNA sequencing is different from microarray and conventional RNA sequencing methods in that it does not assess the abundance of entire transcripts, but rather measures the activity of transcriptional start sites. Perhaps not surprisingly, a study comparing CAGE RNA sequencing directly with microarray and qPCR data found a rather low correlation between the data obtained by these techniques^43^. In our case, we additionally performed a cross-study comparison that likely introduced further bias due to biological differences in the starting material as well as experimental procedures and handling. Additionally, the data sets differed regarding the dosage of the growth factors applied: 1.5 μgml^−1^ in case of VEGF-C156S, 500 ngml^−1^ of wildtype VEGF-C, and 20 ng ml^−1^ VEGF-A. However, these doses reflect the differences in affinity and activity of the VEGFs, and are well within the range of what is commonly used by our lab and others^23,44,45^.

Despite these challenges, we observed the expected upregulation of an overlapping gene set after VEGFR-2 and VEGFR-3 stimulation with our approach, at least early after stimulation (60 min) when the induction of many downstream genes peaked. Furthermore, we identified several genes that were selectively regulated by stimulation of VEGFR-2 or VEGFR-3 only. These differentially regulated genes included the selective VEGF-A target genes RCAN1, ESM1 and ANGPT2. The specific induction of these genes by VEGF-A could also be validated experimentally, supporting the overall validity of our analysis. Selective upregulation of genes by VEGFR-2 might be explained simply by a difference in signal ‘strength’, e.g. due to higher ligand-receptor affinity, or higher kinase activity of the receptor, which are hard to control or manipulate experimentally. However, the fact that we also found a few genes regulated selectively by VEGFR-3 suggests that the quality of the signal downstream of the two receptors differs to some extent, e.g. by activating different kinases which in turn activate different TFs. Interestingly, stimulation with wildtype VEGF-C resulted in a ‘mixed’ response, with some genes being regulated as by VEGF-C156S and others as by VEGF-A.

Of note, the genes selectively induced by VEGFR-3 stimulation included two TFs, SOX18 and KLF4, that both play important roles in LEC function and differentiation. SOX18 is induced in LEC progenitors early during embryogenesis and is required for establishment and maintenance of the LEC phenotype^20^. KLF4 is a direct transcriptional target of MAFB, a TF activated by VEGFR-3 signaling^9^, and may be involved in LEC differentiation by regulating the ‘master’ TF of LEC, PROX1 (ref. 21). Thus, in line with our previous study^9^, the results of our meta-analysis presented here suggest that VEGFR-3, which is predominantly expressed in LECs, exerts a specific function in LEC differentiation, whereas more general processes shared between LECs and BECs such as cell migration and proliferation are regulated by both VEGFR-2 and VEGFR-3 alike.

## Methods

### Gene expression analysis of VEGF-C156S stimulated LEC

CAGE RNA sequencing data of primary human LECs stimulated with VEGF-C156S have been published previously^9,46^. In brief, dermal LECs isolated from the foreskin of 3 individual donors^47^ were starved in EBM medium (Lonza) + 0.2% bovine serum albumine (BSA) over night and were subsequently incubated with 1.5 μgml^−1^ VEGF-C156S. RNA was extracted at 16 different timepoints (0 min, 15 min, 30 min, 45 min, 60 min, 80 min, 100 min, 120 min, 150 min, 180 min, 210 min, 240 min, 300 min, 360 min, 420 min, 480 min) using TRIzol (Thermo Fisher) and subjected to CAGE RNA sequencing by the Fantom5 consortium at the RIKEN Institute (Yokohama, Japan) as described^9^. Raw counts were downloaded from the freely available Fantom5 data repository (http://fantom.gsc.riken.jp/5). CAGE peak expression analysis over the entire time course was done in R, using Bioconductor’s DESeq2 package (version: 1.12.4 (ref. 12)). A multi-factorial model design was used to compare time point 0 min (control) to the other 15 time points after VEGFC156S stimulation, pairing the samples from each individual donor. Lowly expressed CAGE peaks were removed if their raw count sum was=<1. CAGE peaks with a *P*-value<0.05 and a log_2_FC>0.6 or <−0.6 were considered as differentially expressed (DE). MA plots showing the log_2_FC compared to the average expression of the time point-specific CAGE peaks were done using the DESeq2 package. The corresponding genes (501 upregulated, 772 downregulated) are provided in ‘Upregulated Genes after VEGFC156S stimulation’, ‘Downregulated Genes after VEGFC156S stimulation’, Data Citation 1. Heat maps and hierarchical clustering of the up- and downregulated genes were performed using Genesis (Release 1.76, TU Graz, Austria).

### Gene ontology analysis

The DE genes were grouped into early (0 to 120 min), middle (150 to 240 min) and late (300 to 480 min) phase genes. Gene ontology (GO) enrichment analysis for each of these phases was performed using the Gene Ontology Consortium Database (http://geneontology.org, release 22.8.2016). GO biological process (BP) terms and molecular function (MF) terms with an adjusted *P*-value<0.01 were considered significantly enriched. The top 25 enriched GO terms (by fold enrichment) from each category were clustered manually into biologically related themes and are listed in ‘GO_BP analysis’ and ‘GO_MF analysis’, Data Citation 1.

### Gene expression analysis of VEGF-A and VEGF-C stimulated LECs

Generation of gene expression data of primary human LECs treated with VEGF-A and wildtype VEGF-C has been published before^10^. In brief, dermal LECs were starved in EBM + 0.2% BSA over night and were subsequently incubated with 20 ngml^−1^ VEGF-A or 500 ngml^−1^ mature wildtype VEGF-C (both from R&D Systems). RNA was extracted after 0 min (control), 60 min, 240 min, 480 min and 24 h, and subjected to microarray-based gene expression analysis using the Human Genome Survey V2.0 (Applied Biosystems) as described^10^. This data set has been deposited in the gene expression omnibus (GEO) repository under the accession number GSE11228 (Data Citation 2). Normalized data were retrieved and analyzed using the GEOquery package in Bioconductor, comparing the gene expression at the time points 60, 240 and 480 min to the 0 min (control) time point.

### Comparison of VEGF-C156S, wildtype VEGF-C and VEGF-A regulated genes in LECs

Comparison of the VEGF-C156S stimulation time course and the VEGF-A and VEGF-C stimulation time courses was done based on the log_2_FC values at the matching time points 60, 240 and 480 min. To this end, we first removed all ambiguous CAGE peaks and probe sets. In the case of multiple peaks or probe sets associated with one gene, only the peak or probe set with the highest average expression was considered. Next, we used Entrez Gene IDs derived from corresponding annotation tables (Fantom5 annotation table available at http://fantom.gsc.riken.jp/5/, and GPL2986 from the GEO repository) to match the genes in the three data sets. Genes without available Entrez Gene ID in the corresponding annotation tables were additionally annotated using Bioconductor and the org.Hs.eg.db package (version 3.2.3) based on their gene symbol. The final matching of genes in the three data sets based on the Entrez Gene ID was done using R. From 16639 genes represented on the microarray, 10952 (at 60 min), 10951 (at 240 min), and 10989 (at 480 min) could be matched to the CAGE RNA sequencing data in this way. Log_2_FC values of all matched genes were plotted, and linear regression and correlation coefficients (Pearson) were calculated using GraphPad Prism V5. For the 3 time points, we selected genes differentially regulated by VEGF-C156S and VEGF-A if they were strongly regulated by either VEGF-A or VEGF-C156S (*P*<0.05 and log_2_FC>1 or <−1) and if there was at least a four-fold difference in the log_2_FC value after VEGF-C156S stimulation compared to the log_2_FC after VEGF-A stimulation (‘Genes differentially regulated by VEGF-A and VEGF-C156S’, Data Citation 1).

### Validation of selected genes by qPCR

24 h before stimulation, 70000 primary LECs were seeded on collagen type-I coated 12-well plates. Next, LECs were starved over night in EBM + 0.2% BSA and were subsequently treated for 60, 240 or 480 min with 20 ngml^−1^ VEGF-A (Cell Sciences), 500 ngml^−1^ mature wildtype VEGF-C (R&D Systems) or 1.5 μgml^−1^ recombinant VEGF-C156S (kindly provided by Dr. Kari Alitalo, Wihuri Research Center, Helsinki, Finland). Total RNA was isolated using Genezol reagent (Geneaid) and extracted following the manufacturer’s protocol. The concentration of RNA was measured using a NanoDrop. Equal amounts of RNA were reverse transcribed using the High-Capacity cDNA Reverse Transcription Kit (Applied Biosystems). Gene expression of selected genes was quantified by qPCR using FastStart Universal SYBR Green Master (ROX) on a QuantStudio 7 Flex Real-Time PCR System (Applied Biosystems). RPLP0 was used as housekeeping control to normalize Ct values. Relative expression was calculated according to the comparative Ct method. Primer sequences are listed in Table 1.

## Additional Information

**How to cite this article**: Dieterich, L. C. *et al.* Distinct transcriptional responses of lymphatic endothelial cells to VEGFR-3 and VEGFR-2 stimulation. *Sci. Data* 4:170106 doi: 10.1038/sdata.2017.106 (2017).

**Publisher’s note:** Springer Nature remains neutral with regard to jurisdictional claims in published maps and institutional affiliations.

## Supplementary Material

Supplementary Figure 1

## Figures and Tables

**Table 1:** List of qPCR primers.

**Gene**	**Forward**	**Reverse**
RPLP0	CAGATTGGCTACCCAACTGGT	GGGAAGGTGTAATCCGTCTCC
RCAN1	CAGAATAAACTTCAGCAACCC	CCTATGTGTAAGGTCTGAGC
ANGPT2	CAATTATTCAGCGACGTGAG	AAGGGTTACCAAATCCCAC
ESM1	GTATCTGCAAAGACTGTCCCT	TGTCACAGATGCCTGACTG
