# Supplementary material for: Distinct transcriptional responses of lymphatic endothelial cells to VEGFR-3 and VEGFR-2 stimulation
Source: Sci Data. 2017 Aug 29;4:170106. doi: 10.1038/sdata.2017.106 (PMC5574372; doi:10.1038/sdata.2017.106)
Supplement: Supplementary Figure 1 [file sdata2017106-s1.pdf]

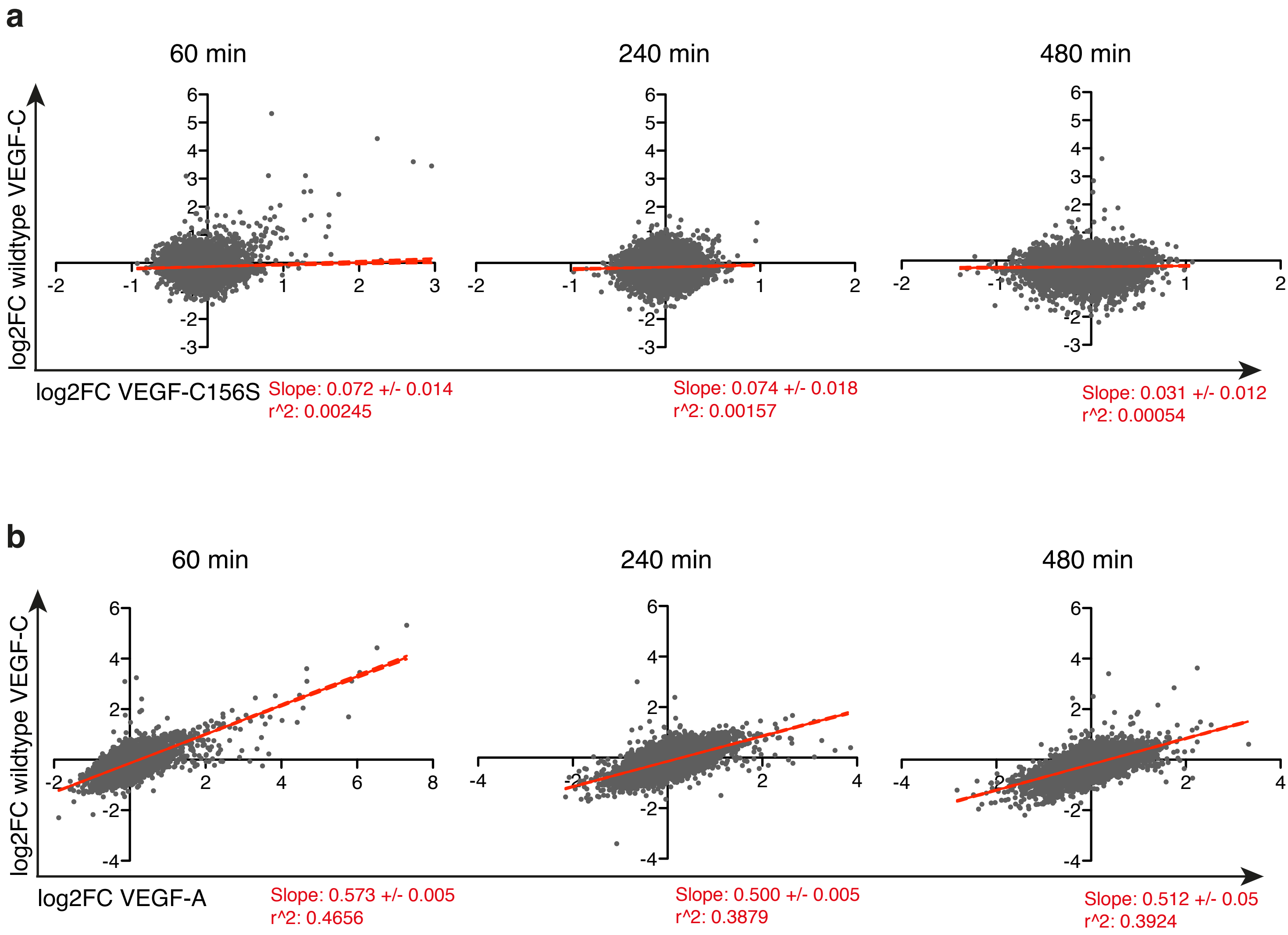

Supplementary Figure 1: Correlation of log2FC of all matchable genes induced by wildtype VEGF-C (y-axes) and VEGF-C156S (x-axes) (a) and by wildtype VEGF-C (y-axes) and VEGF-A (x-axes) (b) in LECs at the 60 min, 240 min, and 480 min time point. Linear regression and Pearson correlation co-efficients are indicated in red.
